# The Social Worker in the Nursing Home: A Systematic Review of the Literature from 2010 to 2020

**DOI:** 10.1093/geroni/igaa057.3508

**Published:** 2020-12-16

**Authors:** Vivian Miller, Tyrone Hamler, Susanny Beltran, Jacquelyn Burns

**Affiliations:** 1 Bowling Green State University, Bowling Green, Ohio, United States; 2 Case Western Reserve University, Cleveland, Ohio, United States; 3 University of Central FLorida, Orlando, Florida, United States

## Abstract

Social workers have an integral role in health care and a long-standing history in nursing homes. The novel pandemic, COVID-19, has disproportionately adversely affected nursing home residents, bringing to the fore the importance of practice in this setting. However, the role of the nursing home social worker is often ambiguous. The objective of this study is to (1) identify existing research studies that discuss the role of social work and nursing facilities, (2) synthesize findings to determine what is most often reported in the literature, (3) present recommendations for practice and implications for research and policy change. This rapid review used the PICO framework and PRISMA guidelines to systematically search for articles published in English between 2010 and 2020 across 11 databases. A final sample of 23 articles met inclusion criteria. Relevant details were extracted from these articles, and the following 3 categories emerged as broadly describing the literature: (1) elements of social work practice in NHs, (2) social worker responsibilities, (3) policy impacts on practice. Findings highlight that the literature on NH social workers is limited and outdated, despite the 2016 federal rule requiring increased consideration for resident quality of life, quality of care, and behavioral health, all features of social work practice. The nursing home social work role warrants greater attention, with an emphasis on how to support residents during the current pandemic to ensure the development and implementation of person-centered care. The literature points to research priorities including involving families and residents in data collection efforts.

